# Internal Brace Augmentation Versus Standard Anterior Cruciate Ligament Reconstruction: A Comparative Clinical Study in Young Active Patients

**DOI:** 10.7759/cureus.105570

**Published:** 2026-03-20

**Authors:** Kanchan K Sabui, Bhaskar Biswas, Suraj Kumar, Shivshankar Roy, Partha P Mandal

**Affiliations:** 1 Orthopaedics, Medical College Kolkata, Kolkata, IND

**Keywords:** anterior cruciate ligament reconstruction, arthroscopy, internal fixators, range of motion, return to sport, treatment outcome

## Abstract

Background and aims: Anterior cruciate ligament (ACL) rupture is a common injury in young, active individuals, and arthroscopic ACL reconstruction (ACLR) is the standard of care. Internal brace augmentation has been proposed to enhance graft protection and potentially facilitate earlier return to sport, but comparative clinical data remain limited. This study aimed to compare early functional outcomes, knee stability, return to sports, and complications between internal brace augmentation ACLR and standard ACLR in patients with ACL rupture.

Methods: This prospective comparative study was conducted at Medical College Kolkata, a tertiary care teaching hospital in Kolkata, India. Seventy patients with unilateral ACL rupture were analyzed: Group A (internal brace augmentation ACLR, n = 40) and Group B (standard ACLR, n = 30). Baseline demographics and injury characteristics were recorded. Functional outcomes (International Knee Documentation Committee (IKDC) and Lysholm scores) were assessed preoperatively and at six months. Knee range of motion (ROM), clinical stability tests (anterior drawer, Lachman, and pivot shift), time to return to sports, and postoperative complications were also evaluated. Between-group comparisons used Mann-Whitney U and chi-square or Fisher’s exact tests, with p < 0.05 considered significant.

Result: Baseline characteristics, including age (27.00 ± 5.60 vs. 26.53 ± 3.07 years; p = 0.638), sex, side, and mechanism of injury, were comparable between groups. Preoperative IKDC scores were similar (49.48 ± 3.19 vs. 49.83 ± 3.19; p = 0.659), improving at six months to 78.98 ± 4.54 in Group A and 77.83 ± 6.72 in Group B (p = 0.858). Preoperative Lysholm scores (54.28 ± 3.29 vs. 54.57 ± 3.07; p = 0.672) improved to 88.88 ± 4.31 and 87.60 ± 6.58, respectively (p = 0.761). At six months, most patients achieved ROM 0-130° or 0-140° (39 (97.5%) Group A, 27 (90.0%) Group B; p = 0.288), and all had negative anterior drawer and Lachman tests; pivot shift was Grade 0 in 30 (75.0%) vs. 20 (66.7%) and Grade 1 in 10 (25.0%) vs. 10 (33.3%) (p = 0.445). Internal brace patients returned to sports significantly earlier (6.38 ± 0.98 vs. 8.20 ± 0.61 months; p < 0.001). Complications were low and comparable: knee stiffness 1 (2.5%) vs. 3 (10.0%) and effusion 3 (7.5%) vs. 2 (6.7%).

Conclusion: Internal brace augmentation ACLR provides early functional outcomes, knee stability, and complication rates comparable to standard ACLR, with the added benefit of a significantly earlier return to sports. These findings support internal brace augmentation as a safe and effective option for young, active ACL rupture patients, while longer-term studies are warranted.

## Introduction

Anterior cruciate ligament (ACL) rupture is one of the most common serious knee injuries in young and active individuals, particularly those participating in pivoting, cutting, and contact sports. Untreated or inadequately managed ACL deficiency can lead to persistent functional instability, reduced activity levels, and an increased risk of secondary meniscal and chondral injury, ultimately predisposing to early osteoarthritis. Over the past two decades, arthroscopic ACL reconstruction (ACLR) using autograft tendon has become the gold standard treatment for restoring knee stability and enabling patients to return to their pre-injury level of activity. Large clinical series from both Western and Indian centers report that ACLR is most frequently performed in young adults, often in the third decade of life, with sports injuries and road traffic trauma being the most common mechanisms of injury [1-4].

Despite substantial advances in surgical techniques, fixation devices, and postoperative rehabilitation, conventional ACLR continues to present several challenges, including residual laxity, delayed return to sport, and a measurable rate of graft failure or revision surgery. In response to these concerns, internal brace ligament augmentation (IBLA) has emerged over the past decade as a potential adjunct to conventional reconstruction. This technique uses high-strength suture tape integrated with the graft to function as a load-sharing construct during the early healing phase. By reducing graft elongation and increasing construct stiffness and failure load, internal brace augmentation may theoretically protect the graft during rehabilitation and permit earlier functional loading without compromising knee stability [1-4].

Clinical evidence supporting internal bracing in ACL surgery has expanded in recent years. Several case series, cohort studies, and systematic reviews have reported satisfactory functional outcomes based on commonly used measures such as the International Knee Documentation Committee (IKDC) score, Lysholm Knee Score, and Knee Injury and Osteoarthritis Outcome Score (KOOS), along with acceptable complication and failure rates. Some comparative studies have suggested trends toward faster functional recovery or earlier return to sports following internal brace-augmented ACLR, although overall stability and functional outcomes are generally comparable to standard ACLR. A randomized controlled trial protocol comparing internal brace-augmented ACLR with conventional ACLR has also been reported, reflecting ongoing uncertainty regarding the true incremental clinical benefit of this technique [1,2,5,6].

Nevertheless, several important gaps remain in the literature. Many existing studies include heterogeneous surgical indications (ACL repair versus reconstruction), different graft types, and variable rehabilitation protocols, which complicate the interpretation of the true role of internal brace augmentation in routine ACLR. In addition, comparative data from Indian and other low- and middle-income settings remain limited despite a growing burden of ACL injuries in these regions. Well-documented prospective comparative studies evaluating patient-reported outcomes, knee stability, return-to-sport timelines, and complications using standardized surgical and rehabilitation protocols are still relatively scarce [1-6].

Against this background, the present study was undertaken to prospectively compare internal brace augmentation ACLR with standard ACLR in young active patients with ACL rupture. The primary objective was to compare early functional outcomes using IKDC and Lysholm scores at six months postoperatively. Secondary outcomes included knee range of motion, clinical stability tests, time to return to sports, and postoperative complications. This study aims to provide preliminary comparative evidence on whether internal brace augmentation offers any early clinical advantage over conventional ACLR without compromising safety.

## Materials and methods

Study design and setting

This was a prospective comparative study conducted at Medical College Kolkata, a tertiary care teaching hospital in Kolkata, India, with a dedicated arthroscopy and sports medicine service. Consecutive patients with symptomatic ACL rupture undergoing primary arthroscopic ACLR were enrolled and allocated to either internal brace augmentation ACLR (Group A) or standard ACLR (Group B). A total of 70 patients were analyzed, with 40 in Group A and 30 in Group B. The study adhered to the principles of the Declaration of Helsinki, and an Institutional Ethics Committee approval was obtained prior to commencement. All participants provided written informed consent before enrolment.

Eligible patients were adults with a clinically and radiologically confirmed complete ACL rupture, indicated for surgical reconstruction after failure of conservative management or in the presence of functional instability. Diagnosis was based on history, physical examination (positive Lachman and/or pivot shift), and MRI findings. Inclusion criteria included age between 18 and 45 years, unilateral ACL injury, and willingness to comply with postoperative rehabilitation and follow-up protocols. Exclusion criteria included multi-ligament knee injuries requiring additional ligament reconstruction, previous surgery on the index knee, advanced radiographic osteoarthritis, associated fractures around the knee, systemic inflammatory joint disease, and contraindications to anesthesia or surgery. Patients with concomitant meniscal tears amenable to partial meniscectomy or repair were not excluded and were treated as required.

Patients were allocated to one of two treatment groups: internal brace augmentation ACLR (Group A) or standard ACLR (Group B). The allocation was determined according to a preoperative plan formulated during surgical scheduling and was based on the operating surgeon’s preference and the availability of the internal brace implant; therefore, randomization was not performed. In cases where the suture tape augmentation device required for internal bracing was unavailable, patients underwent conventional ACLR. All procedures were performed by or under the supervision of the same team of fellowship-trained arthroscopy surgeons. Standardized operative techniques were used within each group to minimize procedural variability and performance bias. In addition, key surgical parameters, including graft type, tunnel positioning, fixation methods, and postoperative rehabilitation protocols, were kept consistent between groups to ensure comparability of the interventions.

Surgical technique

The surgical procedures were performed arthroscopically under spinal or general anesthesia with pneumatic tourniquet control following limb exsanguination. Standard anterolateral (viewing) and anteromedial (working) portals were established, and an accessory anteromedial portal was created when required to facilitate femoral tunnel drilling. A systematic diagnostic arthroscopy was undertaken to confirm ACL rupture and to identify and manage associated intra-articular pathologies, including meniscal tears.

**Figure 1:**
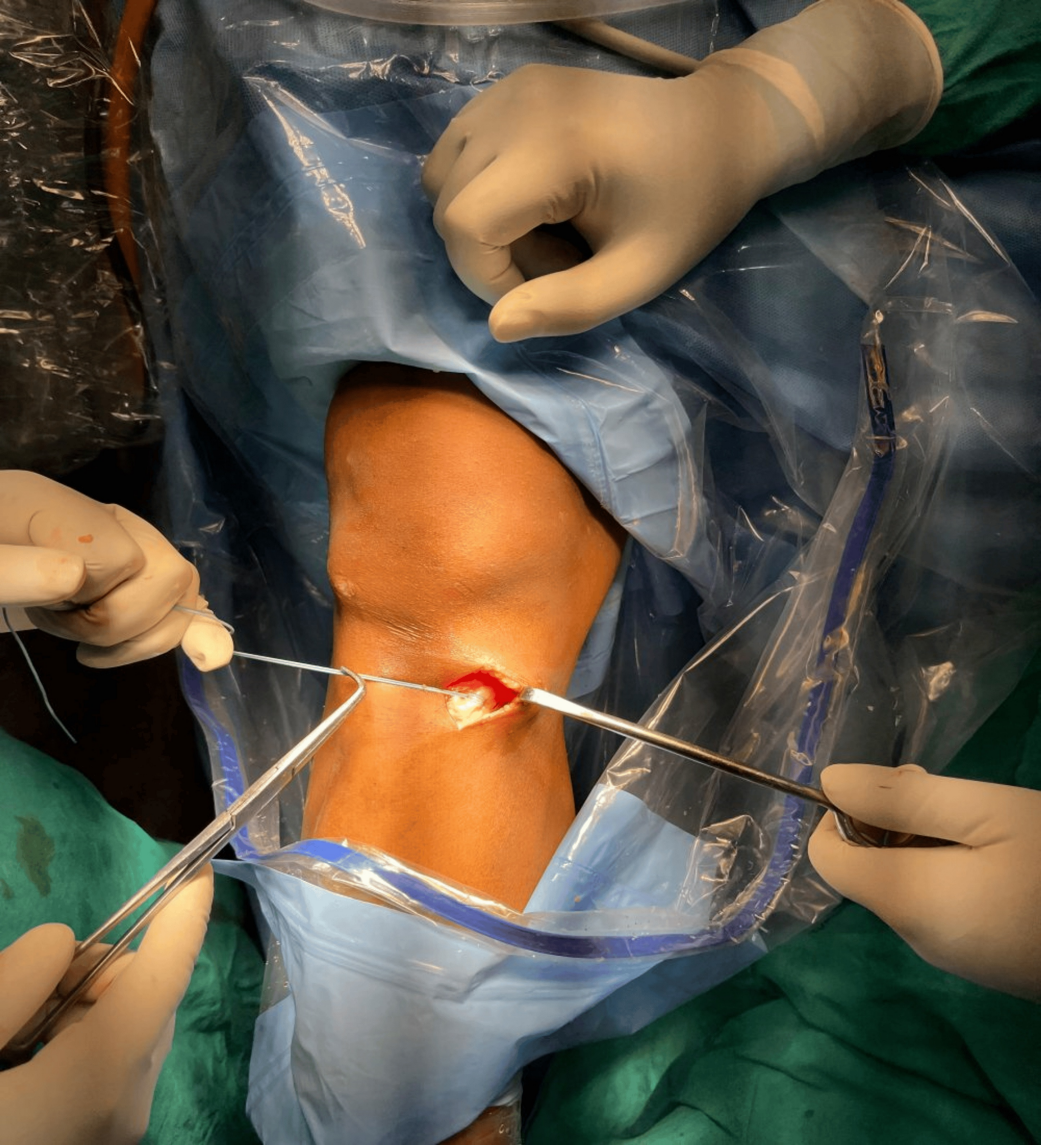
Intraoperative photograph showing an oblique incision over the pes anserinus for harvesting the semitendinosus ± gracilis tendons.

A 3-4 cm oblique incision was made over the pes anserinus to harvest the hamstring graft. The sartorial fascia was incised, and the semitendinosus tendon, with or without the gracilis tendon, was identified and harvested. The graft was prepared as a quadrupled hamstring construct, and both free ends were whip-stitched using high-strength sutures. In our study protocol, a graft diameter of 8 mm was used as the standard for conventional ACLR. For grafts measuring <8 mm in diameter, ACLR augmented with an internal brace was performed to enhance biomechanical strength and reduce the risk of graft failure. In addition, in high-demand or athletic patients, internal brace augmentation was also utilized even when the graft diameter was >8 mm, considering their increased functional requirements and risk of reinjury. The final graft diameter was measured to guide tunnel preparation prior to implantation (Figure 1).

The ACL remnant was debrided, and minimal notchplasty was carried out when necessary to prevent graft impingement. Tibial tunnel preparation was performed using an ACL tibial guide set at 55-60°. Under arthroscopic visualization, a guide pin was inserted at the native tibial footprint of the ACL, followed by sequential reaming according to the measured graft diameter. Femoral tunnel preparation was accomplished using the transportal technique. With the knee flexed to 110-120°, a guide pin was positioned at the anatomic femoral footprint. After confirming accurate placement, the femoral tunnel was reamed to the appropriate depth, and the tunnel length was measured.

The prepared graft was passed through the tibial tunnel into the femoral tunnel using a shuttle suture. Femoral fixation was achieved using either a suspensory fixation device, such as a cortical button, or an interference screw. Tibial fixation was performed with a bioabsorbable or titanium interference screw.

**Figure 2:**
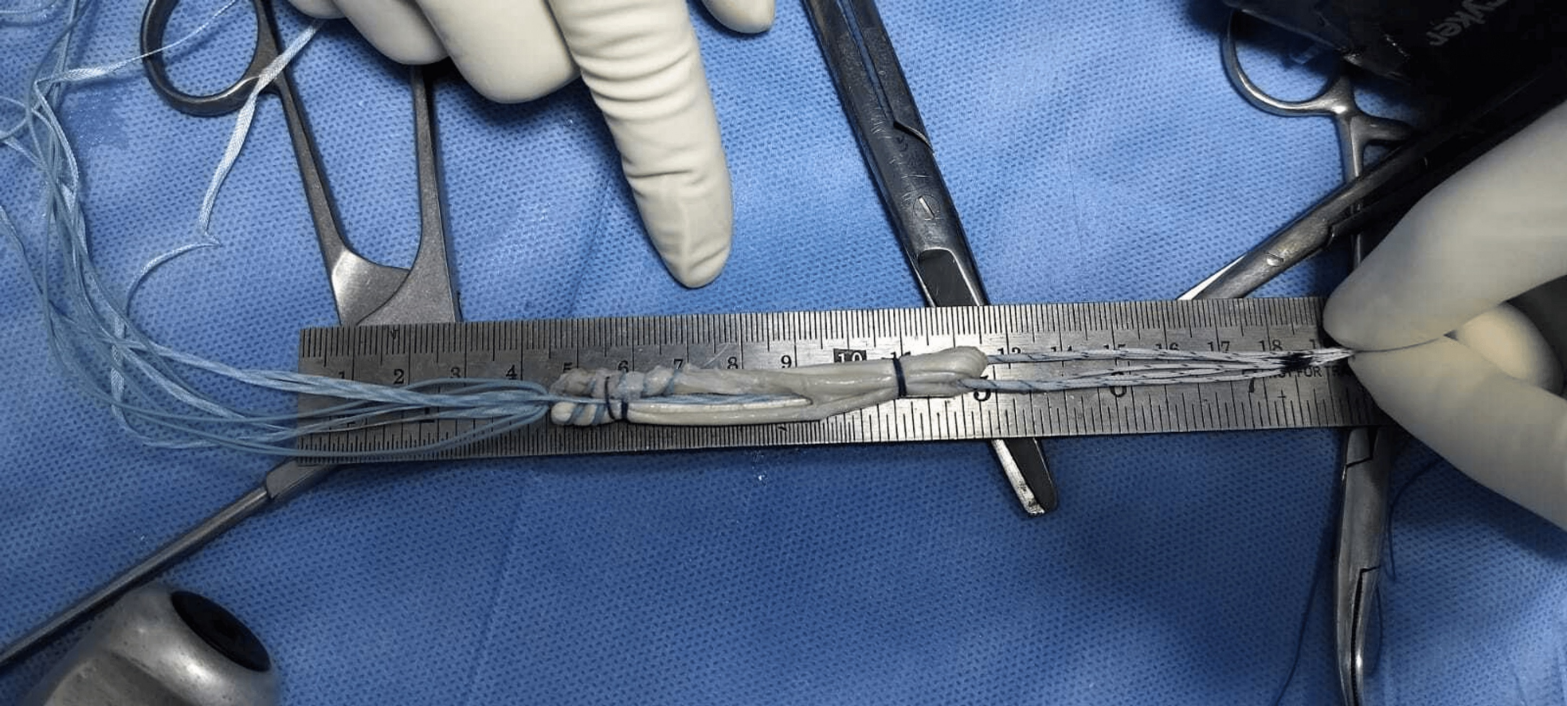
Intraoperative photograph showing suture tape centrally incorporated within the hamstring autograft during graft preparation.

**Figure 3:**
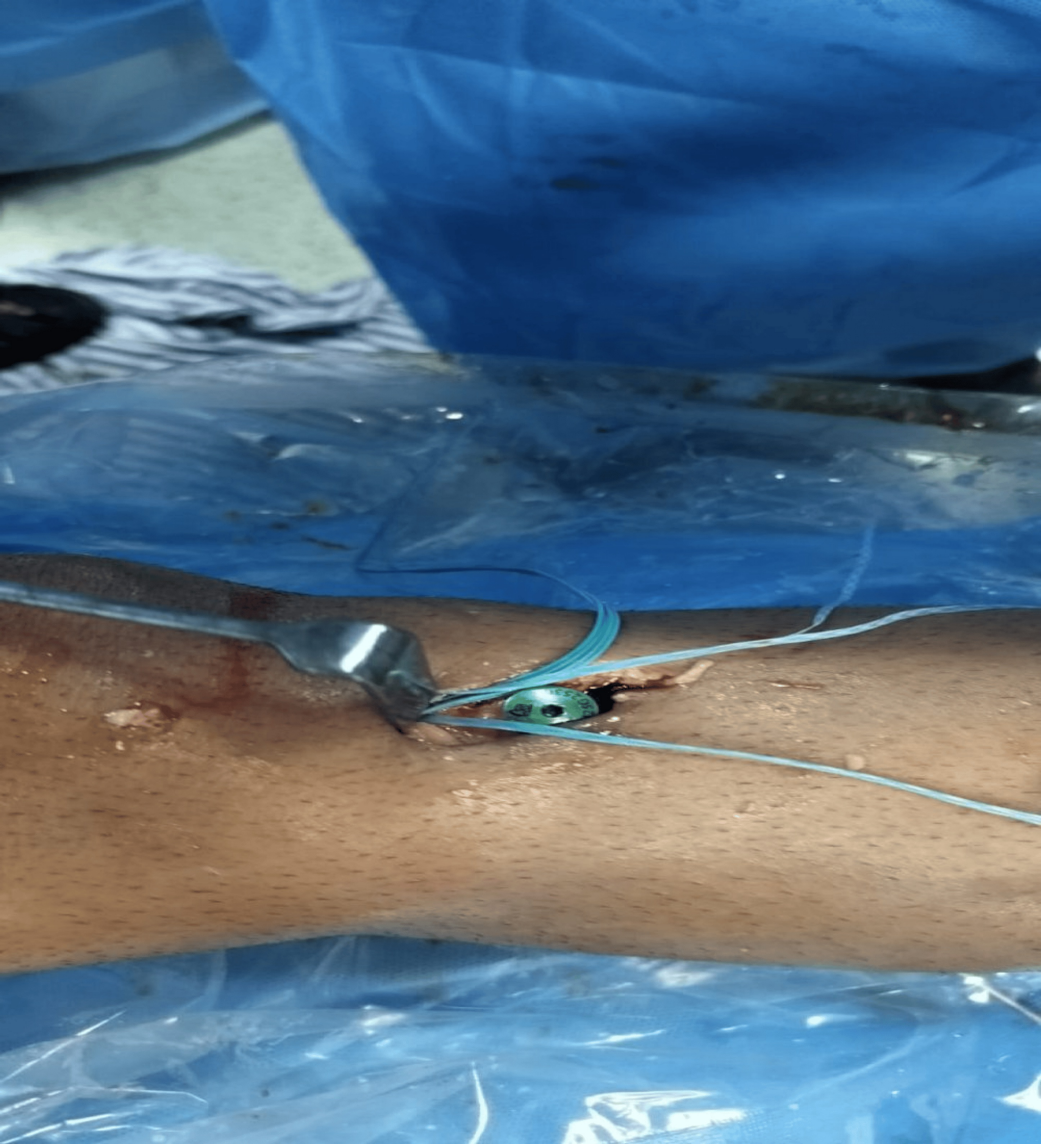
Intraoperative image showing the internal brace tied in full knee extension over the tibial tight rope (TR) fixation device.

**Figure 4:**
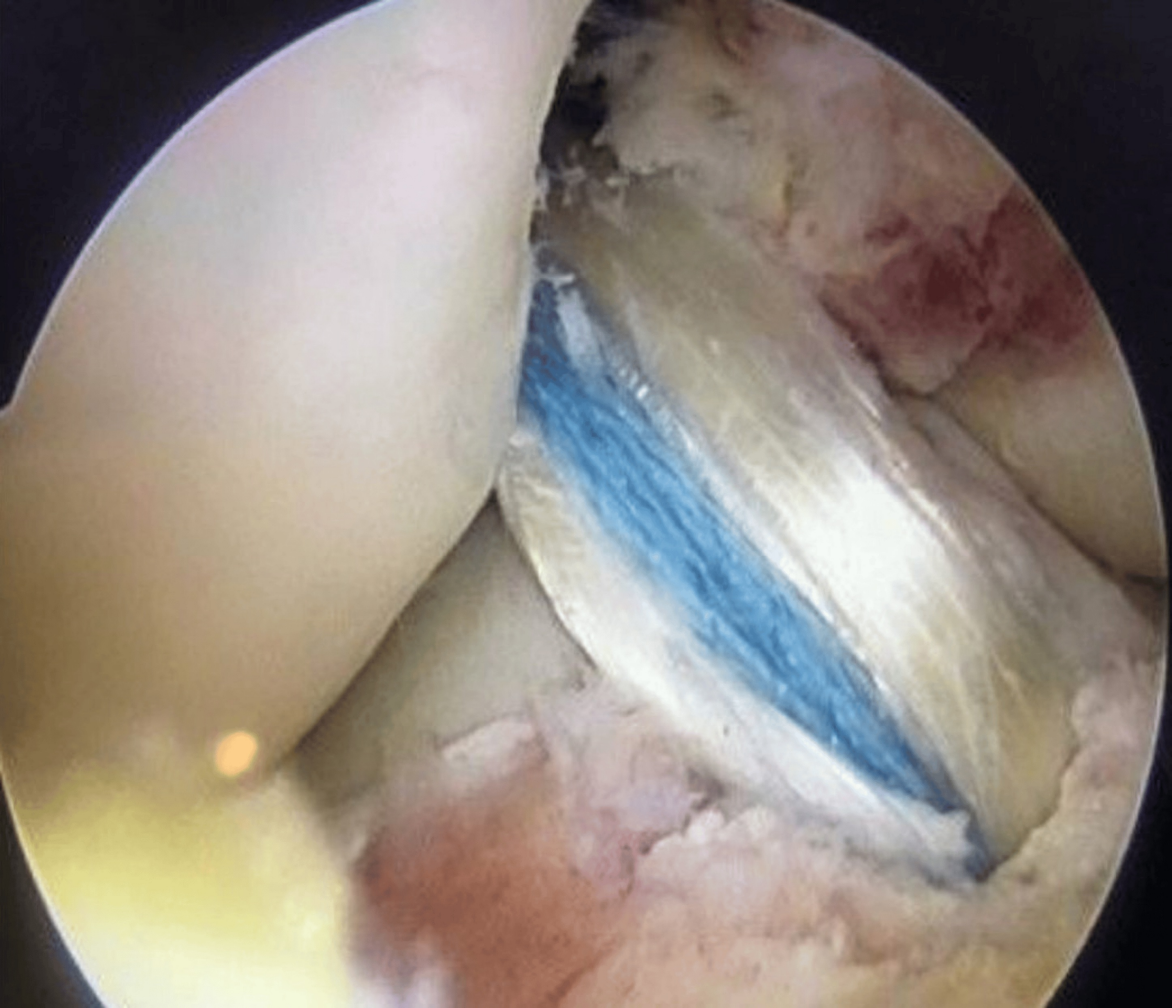
Intraoperative arthroscopic view of the right knee through the anterolateral portal showing a hamstring tendon graft augmented with an internal brace.

In Group A (internal brace augmentation group), a high-strength suture tape was incorporated centrally within the hamstring graft during preparation, ensuring its intragraft positioning to minimize soft tissue irritation. The suture tape was advanced concomitantly with the graft through the femoral and tibial tunnels. In addition to standard graft fixation, the internal brace was independently secured on the tibial side using a suture anchor under appropriate tension. The construct was tensioned in near-full knee extension to function as a secondary stabilizer without over-constraining the joint (Figures 2, 3, 4).

**Figure 5:**
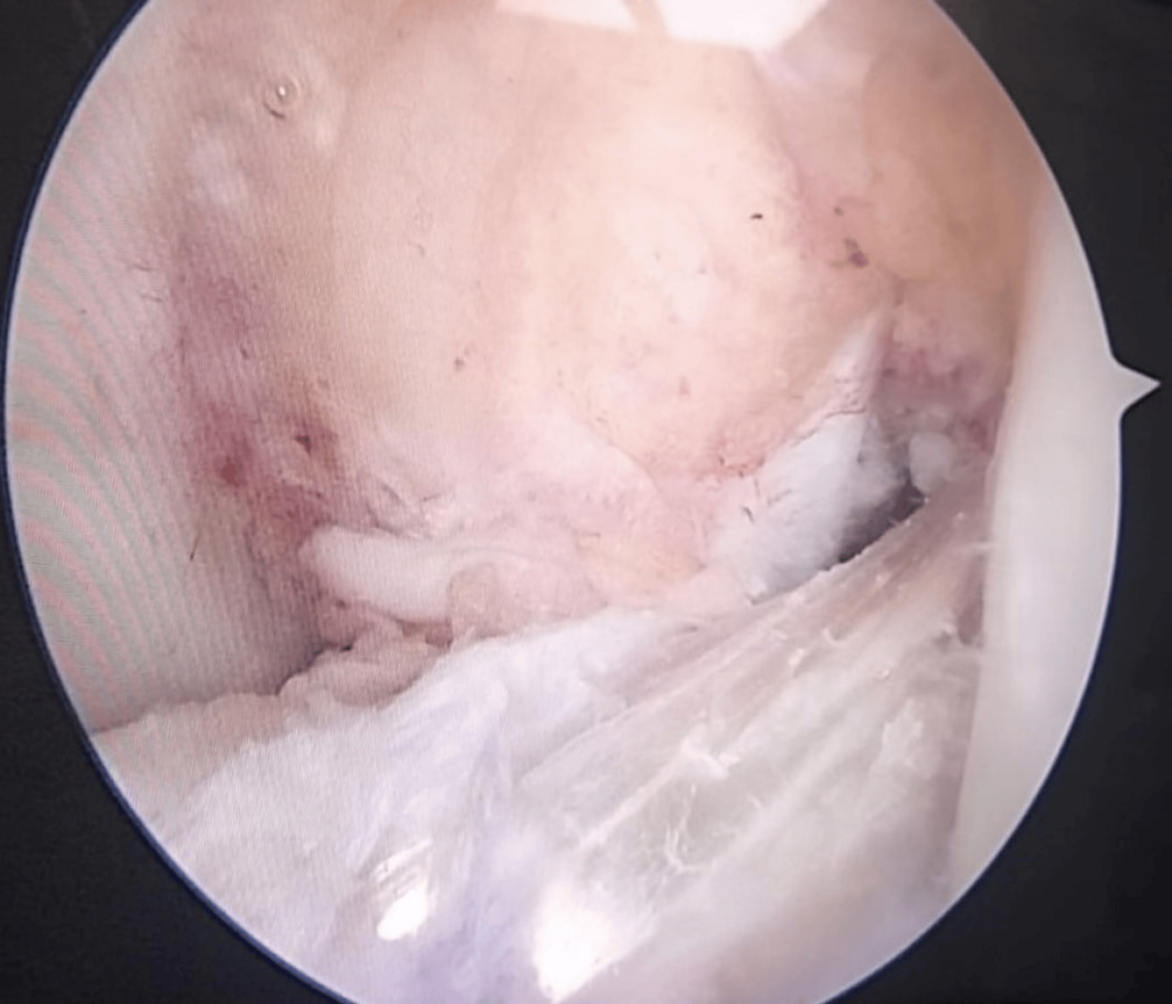
Intraoperative arthroscopic view of the left knee through the anterolateral portal demonstrating conventional ACL reconstruction using a hamstring tendon graft.

In Group B (conventional reconstruction group), ACLR was performed using the same graft type, tunnel placement strategy, and fixation techniques, without internal brace augmentation (Figure 5).

In both groups, the graft was cycled through 20-30 flexion-extension movements to precondition the construct. Final fixation was performed at 20-30° of knee flexion. Graft tensioning parameters and fixation angles were standardized across both groups to ensure procedural consistency.

Postoperative rehabilitation and follow-up

A standardized, protocol-driven postoperative rehabilitation program was implemented for all patients under the supervision of a rehabilitation team to ensure consistency and uniformity of care. The rehabilitation protocol was structured into sequential phases based on time from surgery and functional progression. In the immediate postoperative phase (zero to two weeks), early mobilization was encouraged. Weight-bearing as tolerated was initiated either immediately or within the first few postoperative days. A knee brace was used during ambulation in the initial weeks to provide support. Pain and swelling were managed using cryotherapy and limb elevation. Range-of-motion (ROM) exercises were commenced with the objective of achieving gradual restoration of full knee extension and progressive flexion. Early quadriceps activation was emphasized through isometric quadriceps sets and straight leg raise exercises. During the early rehabilitation phase (two to six weeks), progressive improvement in ROM was targeted, with the goal of achieving near-full flexion. The knee brace was gradually discontinued as quadriceps control improved. Closed-chain strengthening exercises, such as mini-squats and protected-range leg presses, were introduced. Additional strengthening of the hamstring and hip musculature was initiated as tolerated. Gait training was continued to restore a normal walking pattern. The intermediate strengthening phase (six to 12 weeks) focused on advancing closed-chain strengthening exercises. Proprioceptive and neuromuscular training, including balance board activities and single-leg stance exercises, was incorporated. Core strengthening and endurance training were added to the program. Full ROM was maintained while continuing progressive strengthening of the quadriceps and hamstrings. In the advanced training phase (three to six months), patients underwent progressive resistance strengthening. Sport-specific functional drills were introduced in a graded manner, followed by plyometric and agility training when appropriate. Emphasis was placed on enhancing dynamic knee stability and neuromuscular control. Return to running, pivoting, and contact sports was determined based on time elapsed since surgery, clinical examination findings, recovery of muscle strength, functional performance testing, and patient-reported readiness.

Clinical and functional evaluations were conducted at baseline (preoperatively) and at 6 months postoperatively. The primary outcome measures at six months were the International Knee Documentation Committee (IKDC) Subjective Knee Form score and the Lysholm Knee Scoring Scale. The six-month follow-up served as the primary endpoint for outcome analysis. A final assessment at 12 months was performed to evaluate return-to-sport status based on clinical examination, functional recovery, and patient-reported readiness to resume sporting activities. Return to sports activities was permitted only after careful clinical and functional evaluation. The criteria for return to sport included a minimum postoperative period of approximately six months, restoration of full knee range of motion, absence of pain or significant effusion, satisfactory clinical stability on Lachman, anterior drawer, and pivot shift tests, adequate recovery of quadriceps and hamstring strength compared with the contralateral limb, and successful completion of functional rehabilitation exercises. Patient-reported readiness and confidence in knee function were also considered during the final decision to allow return to sporting activities.

Sample size

A total of 70 patients were included in the final analysis, with 40 undergoing internal brace augmentation ACLR and 30 undergoing standard ACLR. The sample size was determined pragmatically based on the number of eligible patients treated during the study period and the feasibility of uniform follow-up, rather than on a formal a priori power calculation. This size was anticipated to allow detection of moderate between-group differences in functional scores and return-to-sport times while providing preliminary comparative data in this setting.

Data collection and management

Preoperative demographic and clinical data, intraoperative details, and postoperative outcome measures were recorded by trained study personnel. Functional scores were obtained using validated language versions of the IKDC and Lysholm questionnaires administered in person during clinic visits. Data were entered into an electronic spreadsheet with appropriate range checks to minimize entry errors, and identifiers were removed for analysis to maintain patient confidentiality.

Statistical analysis

Continuous variables such as age, IKDC scores, Lysholm scores, and time to return to sports were summarized as mean ± standard deviation (SD) and compared between groups using the Mann-Whitney U test because normality assumptions were not met, as reflected in the reporting of U statistics (e.g., U = 560.500 for age; U = 563.00 for preoperative IKDC). Categorical variables, including sex, side of injury, mechanism of injury, ROM categories, stability test results, and complications, were presented as frequencies and percentages and compared using the Chi‑square or Fisher’s exact test. A two-sided p-value <0.05 was considered statistically significant. All statistical analyses were performed using the Statistical Package for the Social Sciences 20.0 (IBM Corp., Armonk, New York, USA).

Ethical considerations

The study protocol was approved by the Institutional Ethics Committee (reference number: MC/KOL/IEC/NON-SPON/1880/05/2023) of the Medical College Kolkata, and all the participants provided written informed consent before surgery. Patient privacy and confidentiality were maintained throughout, and data were used solely for research purposes in anonymized form.

## Results

A total of 70 patients of mean age (26.80 ± 4.664 years) were analyzed between two groups: Group A (internal brace augmentation ACLR) (n = 40) and Group B (standard ACLR) (n = 30). Table 1 summarizes the baseline demographic and injury-related characteristics of the enrolled patients. The mean age was comparable between Group A (internal brace augmentation ACLR) and Group B (standard ACLR), with no statistically significant difference observed (27.00 ± 5.60 years vs. 26.53 ± 3.07 years; U = 560.500, p = 0.638). Gender distribution was similar across both groups, with males constituting approximately two-thirds of participants in each group (27 (67.5%) in Group A and 20 (66.7%) in Group B; χ² = 0.005, p = 0.941). The side of injury was also evenly distributed, with no significant difference between left and right knee involvement across the groups (χ² = 0.172, p = 0.678). Overall, there were no significant differences in baseline characteristics between the two groups, indicating good comparability prior to intervention.

**Table 1:** Age, gender, and side injured of the enrolled patients

Variable	Domain	Group A (n = 40)	Group B (n = 30)	p-value
Age	Mean age	27.00 ± 5.602; mean rank: 34.51	26.53 ± 3.071; mean rank: 36.82	U value: 560.500; p = 0.638
		N (%)	N (%)	
Gender	Male	27 (67.5)	20 (66.7)	$\chi$^2^ = 0.005; p = 0.941
	Female	13 (32.5)	10 (33.3)	
Side injured	Left	18 (45.0)	15 (50.0)	$\chi$^2^ = 0.172; p=0.678
	Right	22 (55.0)	15 (50.0)	

Table 2 compares the mechanism of injury between the two groups. Sports-related injuries were the predominant mechanism in both Group A (internal brace augmentation ACLR) and Group B (standard ACLR), accounting for 28 (70.0%) and 22 (73.3%) of cases, respectively. Road traffic accidents contributed to a smaller proportion of injuries in both groups (12 (30.0%) in Group A and 8 (26.7%) in Group B). No statistically significant difference was observed in the distribution of the mechanism of injury between the groups (χ² = 0.093; p = 0.760), indicating comparable injury patterns at baseline.

**Table 2:** Comparison of mechanism of injury between Group A and Group B

Mechanisms of injury	Group A (n = 40)	Group B (n = 30)	p-value
	N (%)	N (%)	$\chi$^2^ = 0.093; p=0.760
Road traffic accident	12 (30.0)	8 (26.7)	
Sports injury	28 (70.0)	22 (73.3)	

Table 3 presents the comparison of IKDC scores between the two groups at the preoperative stage and at the six-month follow-up. Preoperatively, mean IKDC scores were comparable between Group A (49.48 ± 3.19) and Group B (49.83 ± 3.19), with no statistically significant difference observed (U = 563.00; p = 0.659). At six months post-operatively, both groups demonstrated substantial improvement in IKDC scores, with mean values of 78.98 ± 4.54 in Group A and 77.83 ± 6.72 in Group B. However, the difference between the groups remained statistically non-significant (U = 585.00; p = 0.858), indicating comparable functional outcomes at six months following surgery.

**Table 3:** Comparison of IKDC score at preoperative and six-month follow-ups between the two groups IKDC: International Knee Documentation Committee

IKDC score	Group A (n = 40)		Group B (n = 30)		p-value
	Mean ± SD	Mean rank	Mean ± SD	Mean rank	
Preoperative	49.48±3.194	34.58	49.83±3.185	36.73	U = 563.00; p = 0.659
6 months	78.98±4.538	35.88	77.83±6.721	35.00	U = 585.00; p = 0.858

Table 4 compares the Lysholm scores between the two groups at the preoperative stage and at the six-month follow-up. Preoperatively, the mean Lysholm scores were similar in Group A (54.28 ± 3.29) and Group B (54.57 ± 3.07), with no statistically significant difference between the groups (U = 564.500; p = 0.672). At six months post-operatively, both groups showed marked improvement in Lysholm scores, reaching mean values of 88.88 ± 4.31 in Group A and 87.60 ± 6.58 in Group B. The intergroup difference at follow-up was not statistically significant (U = 574.500; p = 0.761), indicating comparable functional recovery in both treatment groups.

**Table 4:** Comparison of Lysholm scores at preoperative and six-month follow-ups between the two groups

Lysholm score	Group A (n = 40)		Group B (n = 30)		p-value
	Mean ± SD	Mean rank	Mean ± SD	Mean rank	
Preoperative	54.28 ± 3.289	34.61	54.57 ± 3.070	36.14	U = 564.500; p = 0.672
6 months	88.88 ± 4.310	36.68	87.60 ± 6.579	34.65	U = 574.500; p = 0.761

Table 5 compares the postoperative knee ROM at six months between the two groups. The majority of patients in Group A and Group B achieved good functional ROM, with most demonstrating knee flexion ranges of either 0-130° or 0-140°. In Group A, 20 (50.0%) attained a ROM of 0-130°, and 19 (47.5%) achieved 0-140°, whereas in Group B, 11 (36.7%) and 16 (53.3%) patients reached these respective ranges. A small proportion of patients showed restricted ROM (5-100°), observed more frequently in Group B (3 (10.0%)) compared to Group A (1 (2.5%)). However, the overall distribution of postoperative ROM did not differ significantly between the two groups (p = 0.306), indicating comparable knee mobility outcomes at six months.

**Table 5:** Comparison of postoperative range of motion (ROM) between the two groups *Fisher-Freeman-Halton exact test. Data are presented as a number (percentage). P-value calculated using Fisher-Freeman-Halton exact test because the expected cell frequencies were <5 in some cells.

Variable	Degree	Group A (n = 40)	Group B (n = 30)	p-value*
		N (%)	N (%)	p = 0.306
Knee range of movements at six months	0-130 degrees	20 (50.0)	11 (36.7)	
	0-140 degrees	19 (47.5)	16 (53.3)	
	5-100 degrees	1 (2.5)	3 (10.0)	

Table 6 summarizes the clinical stability test outcomes in both groups at follow-up. All patients in Group A and Group B demonstrated negative anterior drawer and Lachman tests, indicating satisfactory anterior knee stability with no intergroup difference. On pivot shift testing, the majority of patients exhibited Grade 0 findings in both groups (30 (75.0%) in Group A and 20 (66.7%) in Group B), while Grade 1 pivot shift was observed in 10 (25.0%) and 10 (33.3%) of patients, respectively. The distribution of pivot shift grades did not differ significantly between the two groups (χ² = 0.583; p = 0.445), suggesting comparable rotational knee stability following both surgical techniques.

**Table 6:** Comparison of clinical test outcomes between Group A and Group B NA: not applicable

Clinical tests	Outcome	Group A (n= 40)	Group B (n = 30)	p-value
		N (%)	N (%)	
Anterior drawer test	Positive	0	0	NA
	Negative	40 (100.0)	30 (100.0)	
Lachman test	Positive	0	0	NA
	Negative	40 (100.0)	30 (100.0)	
Pivot shift test	Grade 0	30 (75.0)	20 (66.7)	$\chi$^2^ = 0.583; p=0.445
	Grade 1	10 (25.0)	10 (33.3)	

Table 7 compares the time to return to sports between the two groups. Patients in Group A (internal brace augmentation ACLR) returned to sports significantly earlier than those in Group B (standard ACLR), with a mean duration of 6.38 ± 0.98 months compared to 8.20 ± 0.61 months, respectively. This difference was statistically highly significant (U = 81.0; p < 0.001), indicating a substantially faster return to sports following internal brace augmentation ACLR.

**Table 7:** Return to sports between the two groups

Variable	Group A (n = 40)		Group B (n = 30)		p-value
	Mean ± SD	Mean rank	Mean ± SD	Mean rank	
Return to sports (months)	6.38±0.98	22.53	8.20±0.610	52.80	U = 81.0; p < 0.001

The comparison of postoperative complications between the two groups showed a low and comparable incidence overall. Knee stiffness was observed in one (2.5%) patient in Group A and three (10.0%) patients in Group B, while joint effusion occurred in three (7.5%) and two (6.7%) patients, respectively. There was no statistically significant difference in the distribution of complications, knee stiffness (p = 0.31), and effusion (p = 1.00) between the groups, indicating similar safety profiles for internal brace augmentation ACLR and standard ACLR (Table 8).

**Table 8:** Complications observed in Group A and Group B *Fisher’s exact test p-values were calculated using Fisher’s exact test due to small expected cell frequencies.

Complications	Group A (n = 40)	Group B (n = 30)	p-value*
	N (%)	N (%)	
Knee stiffness	1 (2.5)	3 (10.0)	0.31
Effusion	3 (7.5)	2 (6.7)	1.00

## Discussion

This comparative study demonstrated that internal brace augmentation ACLR and standard ACLR yielded similar early functional and stability outcomes at six months, but internal bracing was associated with a significantly earlier return to sports without an increase in complications. In this cohort of 70 young adults (mean age 26.80 ± 4.664 years), baseline demographic and injury-related variables were well balanced between groups, supporting internal validity and comparability in line with STROBE recommendations for reporting participant characteristics. Age, sex, side involved, and mechanism of injury did not differ significantly, and sports injuries accounted for about 70% of ACL ruptures in both groups, which is consistent with contemporary ACL epidemiology in athletic populations. Preoperative IKDC and Lysholm scores were virtually identical between groups, indicating similar preoperative functional impairment and reducing the risk of confounding by baseline status [7].

At six months, both internal brace augmentation and standard ACLR produced substantial and clinically meaningful improvements in IKDC and Lysholm scores, with no statistically significant intergroup differences. This aligns with a retrospective comparative series on rehabilitation and functional outcomes after internally braced versus standard ACLR, where patient-reported outcome measures (including KOOS) improved similarly between groups and did not differ significantly at short- to mid-term follow-up. A recent systematic review of IBLA in cruciate ligament surgery likewise found consistent gains in IKDC and Lysholm scores with internal bracing, but generally comparable overall functional outcomes to conventional reconstruction techniques in the short term. Together, these data suggest that internal bracing does not compromise functional recovery and may be functionally equivalent to standard ACLR, at least in the first postoperative year [3,8,9].

Objective stability outcomes in the present study were also comparable; all patients in both groups had negative anterior drawer and Lachman tests, and pivot-shift grades did not differ significantly. These findings mirror observations from clinical series and reviews showing that internal brace augmentation provides at least equivalent anterior and rotational stability compared with standard graft fixation in ACL surgery. Biomechanical experiments provide a mechanistic explanation: suture-tape or internal brace reinforcement increases graft stiffness and ultimate failure load, reduces elongation, and acts as a secondary stabilizer during the vulnerable early healing phase. The comparable stability profile in this study supports the concept that internal bracing can reliably protect the graft without inducing excessive laxity or instability [1,3,8,9,10,11]. The most distinctive and clinically relevant finding was the markedly earlier return to sports in the internal brace group (mean 6.0 months) compared with standard ACLR (8.2 months), with a highly significant difference and large effect size. While time to return to sport is multifactorial and influenced by rehabilitation, psychological readiness, and sport demands, the accelerated timeline observed here is consistent with the hypothesized advantages of internal bracing, namely the ability to permit more confident and earlier loading because the brace shares load with the graft [1,8,11].

Evidence from international cohorts supports this interpretation. A matched analysis comparing ACL repair with an internal brace versus traditional reconstruction showed that, at short-term follow-up, internal brace repair enabled a similar level of sports participation and well-being to classic reconstruction using hamstring or quadriceps autografts. Rehabilitation-focused work has reported that internally braced ACLR patients can follow more aggressive rehabilitation while achieving at least comparable functional outcomes, with trends toward better scores at intermediate time points. Narrative and systematic reviews of IBLA emphasize that the technique facilitates rapid early functional recovery and may shorten the time to activity resumption, although high-quality comparative trials are still emerging [3,8,9,12].

The current findings are also in line with a prospective randomized controlled trial protocol from China comparing internal brace-augmented ACLR with standard ACLR, which specifically hypothesized earlier return to pre-injury activity levels and improved early stability in the internal brace arm. Although the final results of that trial are awaited, its design, outcome selection (IKDC as primary, Lysholm, and KOOS as secondary), and two-year follow-up mirror many elements of the present study, highlighting the global interest in whether internal bracing can translate biomechanical advantages into earlier safe return to sport [1].

From an Indian and regional perspective, early reports and meeting abstracts from Indian centers have described the feasibility of internal brace augmentation and favorable early functional outcomes, echoing the present results, though many remain single-arm or short-term series. These local data, combined with the current comparative findings, suggest that internal bracing is a practical option in high-volume sports-injury settings in India, provided implant availability and cost are acceptable [6]. Postoperative complications were infrequent and not significantly different between groups; knee stiffness and effusion rates were low and comparable, and no graft failures were reported at 6 months. This safety profile is consistent with multiple clinical series and systematic reviews that describe internal brace augmentation as safe, with no signal of increased infection, synovitis, or hardware-related complications in the short term. A systematic review of ACL repair with internal bracing reported an overall failure rate of around 10% over longer follow-up, which is within the range reported for standard ACL procedures, and subjective and clinical stability outcomes were satisfactory [3,5,8,9].

Biomechanical work has raised theoretical concerns about over-constraint or stress shielding, but contemporary reviews conclude that when tensioned appropriately, internal bracing tends to share load rather than fully shield the graft, facilitating biological incorporation while providing early protection. The absence of stiffness or symptomatic over-constraint in this cohort supports careful intraoperative tensioning and reinforces that, when applied correctly, internal bracing can be integrated into routine practice without major safety penalties [9,10,11]. In the internal brace group (n = 40), one patient developed postoperative knee stiffness, with a range of motion of 5°-100°, which was attributed to over-tensioning of the graft (technical error). Additionally, three patients developed significant knee effusion secondary to postoperative synovitis, which was managed conservatively. No other complications, such as foreign body reaction related to the internal brace or clinically significant persistent stiffness, were observed in our cohort. However, we acknowledge certain limitations of our study, including a relatively small sample size and a short follow-up duration of 12 months. These factors may have limited our ability to detect less frequent or late-onset complications associated with internal brace augmentation. Taken together, the present study largely corroborates the international literature in three key ways: Functional outcomes: Similar IKDC and Lysholm improvements between internal brace and standard ACLR agree with retrospective comparative data and early prospective work showing comparable patient-reported outcomes [8,9]. Stability: Equivalent clinical stability aligns with biomechanical and clinical evidence that internal bracing enhances or at least maintains stability versus standard techniques [1,3,10,11]. Safety: Low complication rates resemble those reported in systematic reviews and case series, where serious adverse events directly attributable to the internal brace are rare [5,9].

The main point of potential divergence is the magnitude of difference in return-to-sport time. Some comparative studies of internal bracing in ACL repair or reconstruction describe similar return-to-sport rates rather than a large temporal separation, particularly when rehabilitation is not protocolized for early return. In contrast, the present study demonstrated more than a two-month difference in mean return-to-sport timing, which is clinically important. This discrepancy may reflect differences in surgical indication (repair vs. reconstruction), rehabilitation protocols, cultural or occupational expectations, and surgeon or therapist willingness to clear earlier sport participation when an internal brace is present. Therefore, while the direction of effect (no delay, possible acceleration) is consistent, the magnitude may vary across settings [7,8,12].

Strengths and limitations

This study has several strengths. It employed a prospective comparative design to evaluate outcomes following internal brace augmentation ACLR versus standard ACLR in patients with ACL rupture. The study population was defined using clear inclusion and exclusion criteria, and outcomes were assessed using validated patient-reported measures, including the International Knee Documentation Committee (IKDC) score and Lysholm Knee Score. In addition to functional scores, the study evaluated clinically relevant outcomes such as knee range of motion, clinical stability tests, return to sports, and postoperative complications, providing a comprehensive assessment of early postoperative recovery. Furthermore, all procedures were performed by or under the supervision of fellowship-trained arthroscopy surgeons using standardized surgical techniques and similar rehabilitation protocols, which helped minimize procedural variability between groups.

Several limitations should also be considered. Patient allocation was non-randomized and based on surgeon preference and implant availability, which may introduce potential selection bias despite comparable baseline characteristics between groups. The sample size was relatively small, and no formal a priori power analysis was performed, which may limit the ability to detect smaller differences between groups. In addition, the follow-up period was relatively short, with primary outcome assessment at six months, which restricts evaluation of long-term outcomes such as graft failure, recurrent instability, and revision surgery. The study also relied on clinical stability tests without objective instrumented laxity measurements, such as arthrometer testing, which may limit precision in assessing knee stability. Longer-term follow-up of this cohort is ongoing, and future studies with larger sample sizes, randomized designs, and extended follow-up will be important to further establish the long-term effectiveness and durability of internal brace augmentation in ACLR.

Future research directions and recommendations

Future work should focus on adequately powered, multicenter randomized controlled trials directly comparing internal brace-augmented and standard ACLR with follow-up of at least two to five years to capture re-rupture, revision rates, and long-term functional and radiographic outcomes. Incorporating objective laxity measurements (e.g., instrumented arthrometers), quantitative strength testing, and return-to-sport criteria based on functional test batteries would help standardize outcome assessment and align with current sports-medicine recommendations. High-quality Indian data are particularly needed to clarify cost-effectiveness, implant availability, and applicability in diverse sporting and occupational contexts.

Advanced imaging (MRI-based graft signal, tunnel widening, and integration) and, where feasible, biomechanical or gait analysis could elucidate how internal bracing influences graft maturation, load-sharing, and joint kinematics over time. Subgroup analyses by age, activity level, graft type, and concomitant injuries (e.g., meniscal tears) may identify patients who benefit most from internal brace augmentation. Finally, qualitative and psychological studies addressing patient confidence, perceived knee stability, and fear of reinjury could help explain how internal bracing may support an earlier yet safe return to sports.

In clinical practice, these findings support offering internal brace augmentation ACLR as a valid alternative to standard reconstruction in young, active patients, particularly when earlier return to sport is a priority, while counseling that short-term functional outcomes and stability are likely to be similar between techniques and that robust long-term evidence is still evolving.

## Conclusions

In this comparative study of young adults with ACL rupture, internal brace augmentation ACLR demonstrated early clinical outcomes that were at least equivalent to standard ACLR, with both groups achieving substantial and clinically meaningful improvements in IKDC and Lysholm scores at six months, comparable knee range of motion, and uniformly satisfactory anterior and rotational stability on clinical examination. The absence of significant differences in functional scores, stability tests, and complication rates between the two groups indicates that internal brace augmentation is a safe and effective alternative to conventional ACLR in the short term.

Importantly, patients undergoing internal brace augmentation returned to sports significantly earlier than those treated with standard ACLR, without an associated increase in stiffness, effusion, or other complications, suggesting that the additional mechanical support provided by the internal brace may facilitate a more confident and accelerated yet safe progression of rehabilitation. These findings, which are broadly consistent with emerging international literature on internal brace-supported ACL procedures, support the selective use of internal brace augmentation in young, active patients where expedited return to sport is a key objective, while acknowledging that longer-term comparative data on graft survivorship, re-rupture rates, and osteoarthritic changes are still needed before routine universal adoption can be recommended.
